# Initial Self-Healing Temperatures of Asphalt Mastics Based on Flow Behavior Index

**DOI:** 10.3390/ma11060917

**Published:** 2018-05-29

**Authors:** Chao Li, Shaopeng Wu, Guanyu Tao, Yue Xiao

**Affiliations:** State Key Laboratory of Silicate Materials for Architectures, Wuhan University of Technology, Wuhan 430070, China; lic@whut.edu.cn (C.L.); 254546@whut.edu.cn (G.T.); xiaoy@whut.edu.cn (Y.X.)

**Keywords:** initial self-healing temperature, asphalt mastic, flow behavior index, steel slag, limestone

## Abstract

Increasing temperature is a simple and convenient method to accelerate the self-healing process of bitumen. However, bitumen may not achieve the healing capability at lower temperature, and may be aged if temperature is too high. In addition, the bitumen is mixed with mineral filler and formed as asphalt mastic in asphalt concrete, so it is more accurate to study the initial self-healing from the perspective of asphalt mastic. The primary purpose of this research was to examine the initial self-healing temperature of asphalt mastic, which was determined by the flow behavior index obtained from the flow characteristics. Firstly, the texture and geometry characteristics of two fillers were analyzed, and then the initial self-healing temperature of nine types of asphalt mastic, pure bitumen (PB) and styrene-butadiene-styrene (SBS) modified bitumen were determined by the flow behavior index. Results demonstrate that the average standard deviation of gray-scale texture value of limestone filler (LF) is 21.24% lower than that of steel slag filler (SSF), showing that the steel slag filler has a better particle distribution and geometry characteristics. Also the initial self-healing temperatures of asphalt mastics with 0.2, 0.4 and 0.6 LF-PB volume ratio are 46.5 °C, 47.2 °C and 49.4 °C, which are 1.4 °C, 0.8 °C and 0.4 °C higher than that of asphalt mastics with SSF-PB, but not suitable for the evaluation of asphalt mastic contained SBS modified bitumen because of unique structure and performance of SBS.

## 1. Introduction

Due to the impacts of the natural environment and traffic load, asphalt pavement is prone to generate rutting, cracking, looseness and other deformations during the service life [1,2]. At present, to recover these deformations, many maintenance techniques have been developed, such as slurry seal, micro surfacing, gravel seal and other pavement rehabilitation techniques [3,4,5]. However, these methods are passive maintenance after apparent or more serious damages of the road, not only consuming high maintenance cost and affect the safety of pavement and traffic, but also causing a great waste of resources and environmental pollution. Therefore, it is urgent to develop other advanced preventive maintenance technology to prolong the service life of asphalt concrete.

Asphalt concrete can be recognized as a type of self-healing materials according to several researches [6,7]. During the intermittent period of loading, some of the micro cracks in the asphalt concrete can close/repair themselves automatically, resulting in the recovery of the asphalt pavement performance [8,9]. The existing study suggests that the self-healing capacity of bitumen is associated with the temperature variation closely, while in higher temperature, the elastic recovery, viscous flow and molecular diffusion of bitumen can be accelerated to heal the cracks [6,10,11]. Nowadays, researchers have developed so many technics to realize the increase of temperature, such as induction heating, microwave heating, infrared heating and so on. So it still needs efforts to find suitable temperature to conduct heating process. Bituminous materials may not achieve the healing capability if temperature is below suitable self-healing temperature, also may be aged or deteriorate the pavement performance if temperature is too high. According to the viscosity and flow characteristics of bitumen, researchers can select suitable temperature to be the initial self-healing temperature, and guide the maintenance work.

However, in the asphalt concrete, the bitumen is mixed with mineral filler and formed as asphalt mastic, which finally endows the asphalt concrete with self-healing properties, so it can be considered as a more promising and appropriate perspective to study the viscosity and flow characteristics of asphalt mastic to determine the initial self-healing temperature. According to the former researches, asphalt materials could be recognized as a viscoelastic material and act like a Newtonian liquid at high temperature [12]. The Newtonian flow characteristic can be fitted through the following power law relationship [13,14]:

(1) $$\eta^{*}=m{\left|\omega \right|}^{n-1}$$

where:

*ω* represents frequency;
η* represents complex viscosity;
*m* and *n* represents the fitting parameters.

The dimensionless parameter *n* is also called the flow behavior index. The measured material corresponds to a Newtonian fluid while *n* equals 1.0, and appears a higher degree of pseudoplastic properties while *n* is less than 1. If *n* transfers in the variation from 0.9 to 1.0, asphalt mastic can be recognized as near-Newtonian liquid which processes flow characteristic [15]. The temperature when *n* equals 0.9 was confirmed as the initial self-healing temperature in this research. In this paper, limestone filler and steel slag filler were blended with pure bitumen and SBS (styrene-butadiene-styrene) modified bitumen by different filler-bitumen volume ratio to form nine types of asphalt mastic. Firstly, the texture and geometry characteristics of two fillers were analyzed in this research. Secondly, the initial self-healing temperature of nine types of asphalt mastic, pure bitumen and SBS modified bitumen were determined by the flow behavior index. Research findings can be beneficial to maintenance work by selecting suitable temperature based on the type of filler, bitumen and filler-bitumen ration, so that contributing to save energy and reduce energy consumption. In addition, selecting suitable temperature can delay the aging and prolong the service life of bituminous materials.

## 2. Materials and Methods

### 2.1. Raw Materials

The 60/80 penetration graded pure bitumen (PB) and styrene-butadiene-styrene (SBS) modified bitumen mixed by 60/80 penetration graded pure bitumen and 4.5% dosage of SBS particles which supplied by Guochuang Co., Ltd., Wuhan, Hubei, China, were applied in this research. Limestone filler (LF) was obtained from Agoura Stone Processing Factory, Inner Mongolia, China. While according to former research [16], steel slag filler (SSF) was prepared by milling raw Basic Oxygen Furnace (BOF) steel slags supplied by Wuhan Iron and Steel, Wuhan, China, whose original particle size was 9.5–13.2 mm. The basic properties of raw materials were concluded in Table 1.

### 2.2. Experimental Methods

#### 2.2.1. Preparation of Asphalt Mastic

According to the previous studies and results shown in Table 1 [16], the density of steel slag is about 25% higher than that of limestone, if added different filler by the same weight ratio and it may cause the volume distinction of asphalt mastics, so volume control method was used to consistently maintain the same volume composition of different asphalt mastics. In this research, nine types of asphalt mastic were prepared and could be divided into three groups: asphalt mastic contained limestone filler and pure bitumen was named as LF-PB, asphalt mastic contained steel slag filler and pure bitumen was named as SSF-PB, asphalt mastic contained limestone filler and SBS modified bitumen was named as LF-SMB. Each group included three types of asphalt mastic whose filler-volume ratio was 0.2, 0.4 and 0.6. The control group which only contained pure bitumen and SBS modified bitumen were also included. To obtain different types of asphalt mastics, bitumen was firstly heated in sample vessel, which was placed in oil bath pan. Meanwhile the oil bath pan was heated by an electric furnace and a temperature sensor in the sample vessel could guarantee that the modification was constantly kept at 160 °C together with the control of thermostat. Maintaining the shearing rate of 1500 rpm (revolutions per minute) for 3 min through the whole blending, a high-speed shearing machine was applied to ensure homogeneous dispersion of fillers in the bitumen.

#### 2.2.2. Texture Distinction 

In previous studies, the scan electronic microscope (SEM) image of steel slag showed a little difference compared to limestone [17], but the comparison was depended on personal subjective judgment through visual observation and lacked of theoretical foundation. Subdivided into different distinct areas which have homogeneity among them, SEM images can represent the texture diversity of analyzed materials. With Matlab software (version 2016a, MathWorks, MA, USA), the SEM image can be transformed into black and white image that is consisted with a gray scale range of different pixels vary from 0 (darker) to 255 (brighter). The pixel diversity of different gray scale can reflect the texture distinction of material. Smooth texture of materials has little difference in the number of different gray scale’s pixels, and tough texture has obvious difference in the number of different gray scale’s pixels [18]. Finally, we named the gray-scale level as gray-scale texture value, and through Matlab software to run statistical analysis, like average, variance and standard deviation values of pixels of each gray-scale texture value to quantify the fluctuation level of texture distinction accurately.

The texture distinctions of limestone and steel slag fillers were studied by a JSM-5610LV Scan Electronic Microscope manufactured by JEOL, Tokyo, Japan. The resolution of SEM in the high-vacuum and low-vacuum mode was 3.0 nm and 4.0 nm separately. Magnification of 18–300,000× and 100,000× was adopted in this research.

#### 2.2.3. Geometry Characteristics

Barrett [19] pointed that particle geometry of aggregates could be described in three independent properties: surface texture, form and angularity. The surface texture has been evaluated in Section 3.1, therefore the differences of form and angularity named as geometry characteristics were analyzed by aggregate imaging system (AIMS).

**Figure 1:**
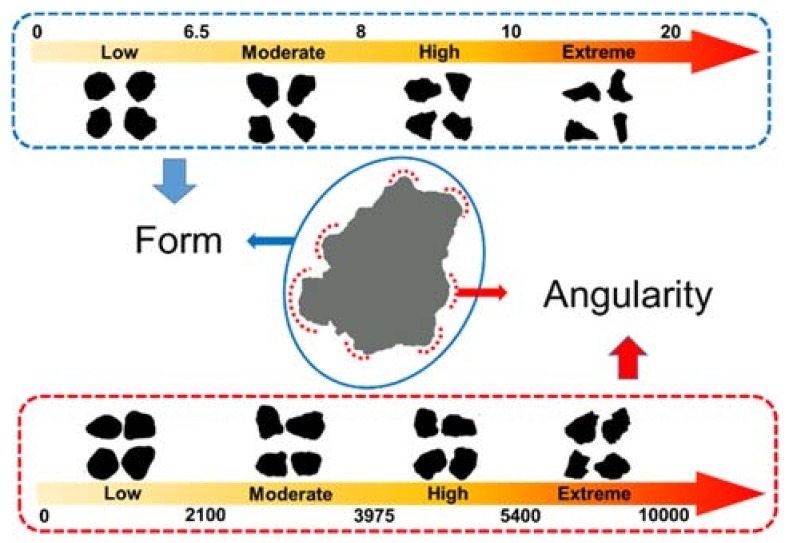
The schematic diagram of aggregate geometry characteristics.

As shown in Figure 1, form represents variations in the proportions of a particle. For the tested filler, the values of form 2D which were analyzed from the relative form of two-dimensional images were used to quantify form distinctions. According to Masad [20], the form 2D was obtained by using incremental change in the particle radius and expressed in the following equation:

(2) $$Form\;2D=\overset{\theta =360-\Delta \theta }{\underset{\theta =0}{\sum }}\frac{\left|R_{\theta +\Delta \theta }-R_{\theta }\right|}{R_{\theta }}$$

In the equation, where *R_θ_* is the radius of the particle at an angle of *θ*, and *Δθ* is the incremental difference in the angle, which is taken to be 4°. The values of form 2D range from 0 to 20, and can be divided into four levels: low (0–6.5, circular), moderate (6.5–8, semi-circular), high (8–10, semi-elongated), extreme (10–20, elongated). The closer the form 2D is to 0, the closer the particle resembles a perfect circle.

In the case of angularity index, Masad [20] developed the radius method which measures the difference between the particle radius in a certain direction and that of an equivalent ellipse, the calculation was on the basis of following equation:

(3) $$Angularity\;Index=\overset{355}{\underset{\theta =0}{\sum }}\frac{\left|R_{\theta }-R_{EE\theta }\right|}{R_{EE\theta }}$$

In the equation, *R_θ_* is the radius of the particle at an angle of *θ*, and *R_EEθ_* is the radius of the equivalent ellipse at an angle of *θ*. The equivalent ellipse has the same aspect ratio of the particle but has no angularity (smooth with no sharp corners). Normalization of the aspect ratio can minimize the effect of form on the angularity index [20]. The values of angularity index range from 0 to 10,000, and can also be divided into four levels: low (0–2100, rounded), moderate (2100–3975, sub-rounded), high (3975–5400, sub-angular), extreme (5400–10,000, angular). The closer the angularity index is to 0, the closer the particle resembles to be rounded.

The geometry characteristics of two fillers were characterized by the AFA2 aggregate imaging system (AIMS), manufactured by PINE, Washington, DC, USA. AIMS captures images of aggregates at different resolutions through a simple setup that consists of one camera and two different types of lighting schemes [21]. The image acquisition setup is configured to capture a typical image of 640 by 480 pixels at these resolutions in order to analyze various sizes of aggregates [22]. Different types of fillers were firstly sieved to select particles which were larger or equal to 0.075 mm, and about 150 particles were analyzed for form 2D and angularity values using black and white images, captured by backlighting under the dedicated sample tray (200#).

#### 2.2.4. Initial Self-Healing Temperature

As the self-healing procedure of asphalt materials depends on temperature, therefore infiltration, dispersion and other thermodynamic motions of bitumen molecules are suffocated at lower temperature, resulting in the resistance to self-healing procedure. However, if temperature achieves the initial self-healing temperature, the molecules can heal the generated cracks more easily and quickly. In the wake of the temperature variation, asphalt mastic could be recognized as a viscoelastic material and act like a Newtonian liquid at high temperature [12]. According to Equation (1), shown in the introduction section, the initial self-healing temperature could be calculated by the relationship between frequency and complex viscosity. In this research, the relationship was obtained through the frequency sweep analysis measured by dynamic shear rheometer (DSR, Anton Paar, Vienna, Austria), which was performed at a dynamic frequency from 0.01 Hz to 10 Hz under different fixed temperature conditions (30 °C, 40 °C, 50 °C and 60 °C). All asphalt mastics were placed on a parallel plate geometry whose diameter was 25 mm, and the thickness of samples was 1 mm.

## 3. Results and Discussions

### 3.1. Texture Distinction

**Figure 2:**
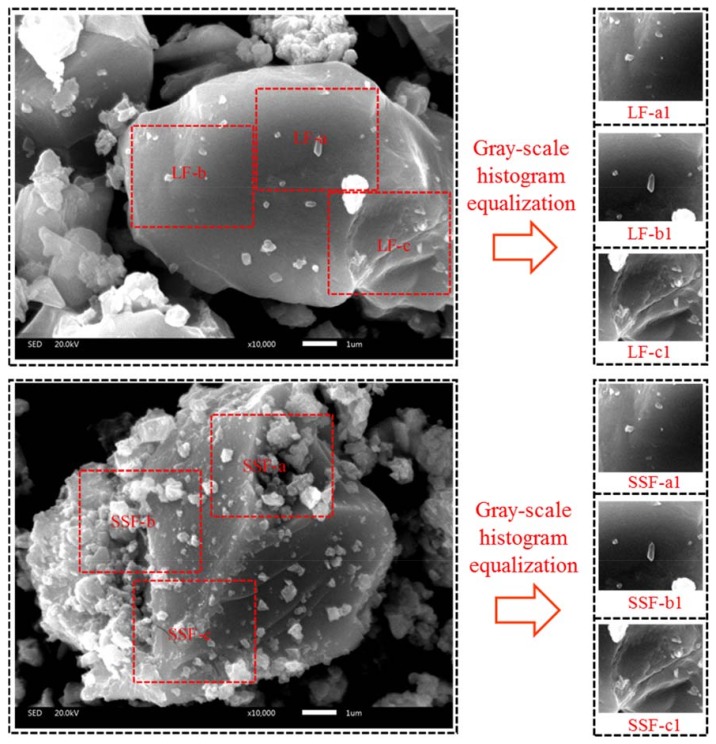
SEM images and gray-scale histogram equalization results of selected areas.

In this paper, three main areas of different fillers in SEM images (as shown in Figure 2) were firstly transformed into gray scale images through gray-scale histogram equalization by Matlab software, which could extend the dynamic range of pixel values to improve the contrast and definition of images. Then the software plotted the gray scale histogram which showed the difference in the number of different gray scale’s pixels. Finally, the standard deviation value of pixel numbers was applied to quantify the texture distinction of two fillers accurately.

**Figure 3:**
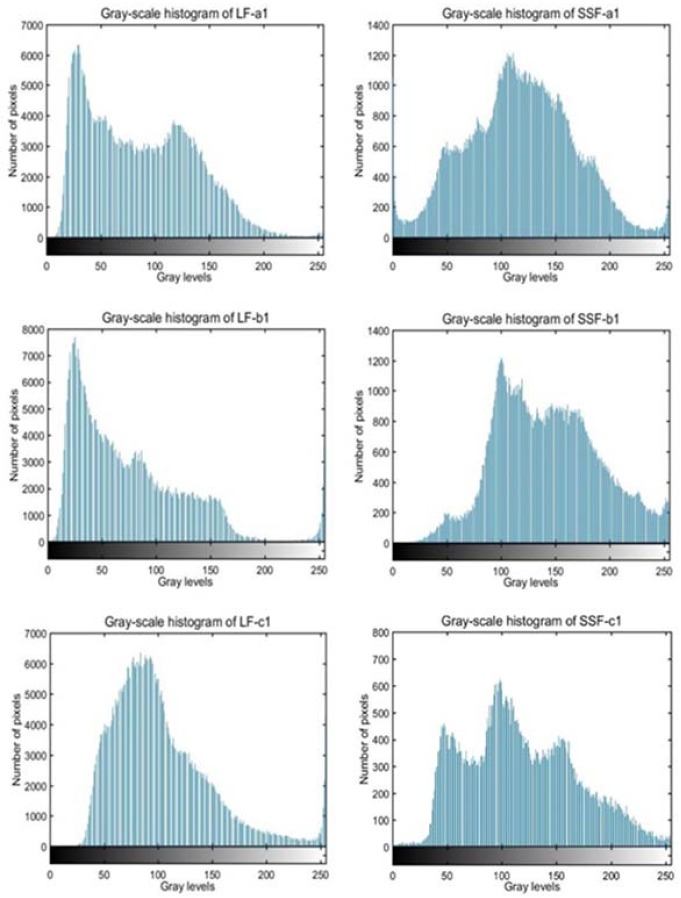
Gray-scale histogram of different areas in two fillers.

Figure 2 shows the SEM images and gray-scale histogram equalization results of selected areas. It is clear to see that particles of steel slag filler assemble together with holes and are tougher than limestone filler. Figure 3 illustrates the gray-scale histogram of different areas in two fillers. The gradient colored from black to white over horizontal axis represents the gray-scale texture value from 0 to 255, while darker color means lower gray level and brighter color means higher gray level. It can be seen that the distributions of limestone filler’s gray-scale texture value are mainly ranging from 0 to 150, and lack pixels in the higher gray-scale texture value. In contrast, the distributions of steel slag filler’s gray-scale texture value are more balanced, and mainly centralized from 50 to 200. It can be estimated initially that steel slag filler has a wider pixel diversity of different gray-scale texture value than limestone filler, which can reflect coarser texture distinction. Table 2 shows the statistical analysis of gray scale histogram. Although the average gray-scale texture values of two fillers have little difference and all ranges from 110 pixels to 150 pixels, the variance and standard deviation values shows significant differences. The variance value of LF is around 1500 while the value of SSF is around 2500, causing that the average standard deviation value of three areas in LF is 21.24% lower than that of SSF. Such differences indicate that SSF with irregular surface texture is coarser than LF which authenticated in the basis of numerical value and statistical analysis.

### 3.2. Geometry Characteristics

**Figure 4:**
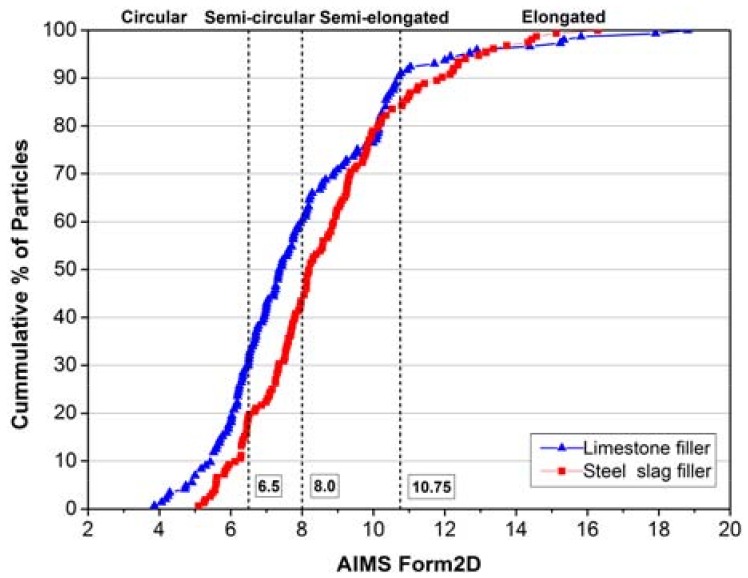
The AIMS form 2D of limestone filler and steel slag filler.

**Figure 5:**
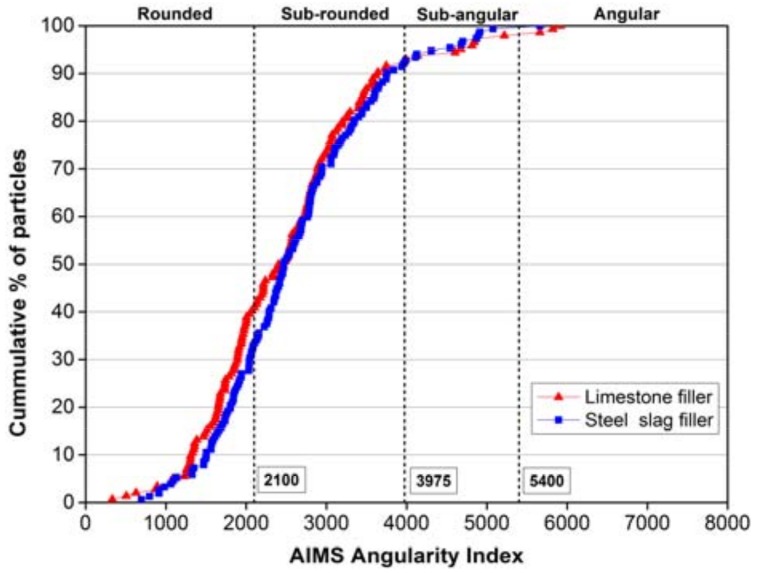
The AIMS angularity Index of limestone filler and steel slag filler.

Figure 4 and Figure 5 illustrate the curves of form 2D and angularity index. It is clearly to see that limestone filler has a wider range than that of steel slag filler both in the form 2D and angularity index curves, representing that limestone is easier to be inhomogeneous and has huge distinctions in form and angularity. Because of more rigid and reliable structure, steel slag can be crushed uniformly and has fewer incidents in the happening of circular, elongated, rounded or angular particles.

Table 3 shows the specific distribution and statistical results of tested form 2D and angularity index. For the values of form 2D, the limestone filler accounts for more percentages than steel slag filler at the low and moderate level, presenting more circular and semi-circular particles. However, steel slag filler processes a higher proportion at high and extreme level, presenting more semi-elongated and elongated particles. The standard deviation of limestone filler is 14.56% higher than that of steel slag filler, demonstrating the former opinion that limestone filler has a wider distribution after being crushed, and the average values present that two fillers are both at the high level but steel slag filler shows better form 2D value than limestone filler. With regard to angularity index, two fillers are distributed rarely at high and extreme level. Although the average values of two fillers are almost the same, but the whole distribution and standard deviation still figure out that limestone filler has a wider distribution, presenting more percentages in the rounded and angular categories, and steel slag filler has a better particle distribution and shape property. Former researches have proved that better geometry characteristics can be beneficial to the cohesion between filler and bitumen [23,24,25]. So under the same deformation factors like sunshine, loading or rainfall, the mastic contained steel slag filler can process better pavement performance, and avoid earlier preventive maintenance than the mastic contained limestone filler.

### 3.3. Initial Self-Healing Temperature of Different Asphalt Mastics

#### 3.3.1. Asphalt Mastic Contained Pure Bitumen and Limestone Filler

**Figure 6:**
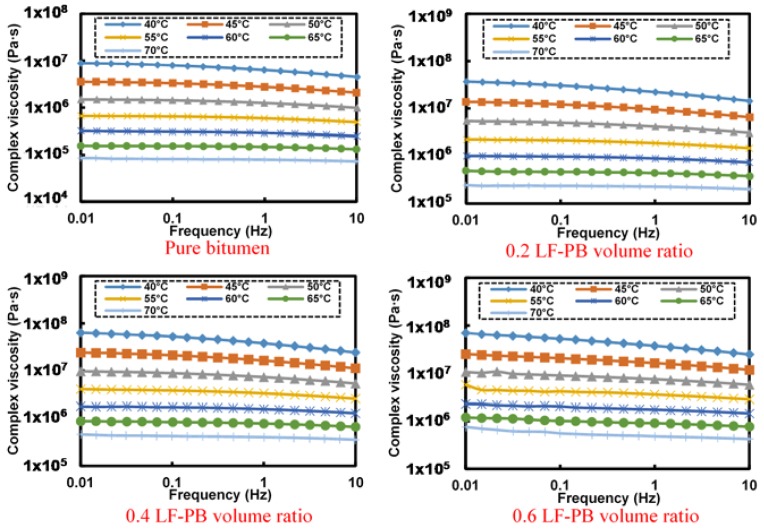
Frequency-complex viscosity relationship of different LF-PB asphalt mastics.

Figure 6 presents the frequency-complex viscosity relationship of LF-PB asphalt mastics. In general, the complex viscosity decreases obviously along with the augment of frequency at 40 °C, but the tendency towards to be flat and smooth as the temperature rises. The curve of frequency-complex viscosity even becomes a horizontal line at 60 °C, which represents that the complex viscosity remains the same with the change of frequency.

Based on the results of Figure 6, Table 4 concludes the fitting results of LF-PB asphalt mastic’s flow behavior index at different temperature. In summary, it can be seen that the flow behavior indexes of all analyzed asphalt mastics show a growing tendency gradually along with the increasing temperature, representing that the asphalt mastics become Newtonian fluid gradually in higher temperature. The flow behavior index of the pure bitumen increases from 0.901 at 40 °C to 0.985 at 70 °C. Existing a little lower value while adding filler, the variation ranges of flow behavior index for asphalt mastics with 0.2, 0.4 and 0.6 LF-PB volume ratio are from 0.863 at 40 °C to 0.979 at 70 °C, 0.860 at 40 °C to 0.969 at 70 °C, and 0.852 at 40 °C to 0.940 at 70 °C, respectively.

#### 3.3.2. Asphalt Mastic Contained Pure Bitumen and Steel Slag Filler

**Figure 7:**
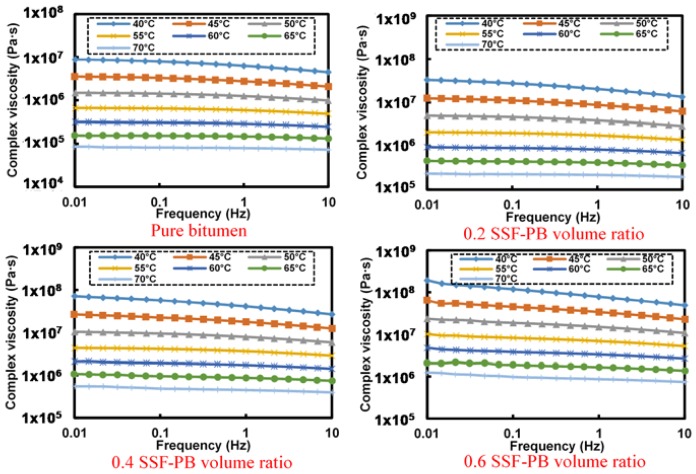
Frequency-complex viscosity relationship of different SSF-PB asphalt mastics.

Figure 7 shows the frequency-complex viscosity relationship of SSF-PB asphalt mastics with different filler-bitumen volume ratio. The general variation tendency of curves is consistent with that of LF-PB asphalt mastics, but still demonstrates a little difference that all SSF-PB asphalt mastics behave higher complex modulus than those of corresponding LF-PB asphalt mastics, which means that SSF-PB asphalt mastics are more superior to resist high-temperature deformation.

Based on the results of Figure 7, Table 5 concludes the fitting results of SSF-PB asphalt mastic’s flow behavior index at different temperatures. With regard to SSF asphalt mastics, the flow behavior indexes become higher than that of LF-PB asphalt mastic. The variation ranges of asphalt mastics with 0.2, 0.4 and 0.6 SSF-PB volume ratio are from 0.869 at 40 °C to 0.978 at 70 °C, 0.857 at 40 °C to 0.953 at 70 °C and 0.828 at 40 °C to 0.945 at 70 °C, respectively.

#### 3.3.3. Asphalt Mastic Contained SBS Modified Bitumen and Limestone Filler

**Figure 8:**
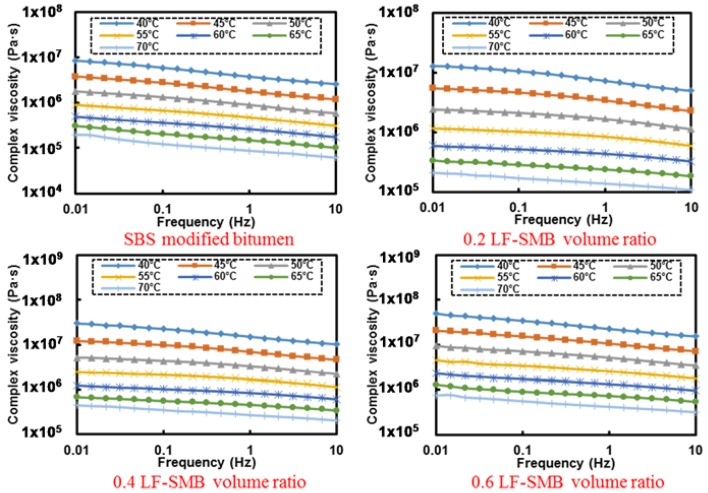
Frequency-complex viscosity relationship of different LF-SMB asphalt mastics.

Figure 8 presents the frequency-complex viscosity relationship of LF-SMB asphalt mastics with different filler-bitumen volume ratio. In contrast to former curves of pure bitumen’s flow behavior indexes, the general tendency is the same that the complex viscosity decreases along with the augment of frequency. However, the curves at 60 °C are not as smooth as former and they still behave a shape dropping trend, meaning that all LF-SMB asphalt mastics possess a distinct flow behavior at different temperature conditions.

**Figure 9:**
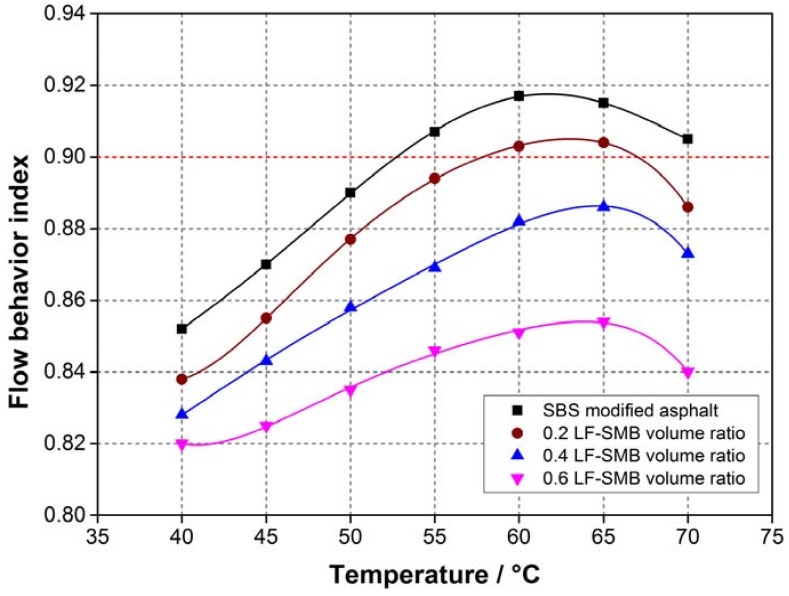
Flow behavior index of different LF-SMB asphalt mastics at different temperatures.

Based on the results of Figure 8, Table 6 illustrates the fitting results of LF-SMB asphalt mastic’s flow behavior index at different temperature. Figure 9 shows the curves of flow behavior index. In contrast to the former analyzed results of LF-PB and SSF-PB asphalt mastics, the flow behavior index curves of all LF-SMB asphalt mastics present different phenomena. Firstly, all the initial flow behavior indexes are lower than these of LF-PB and SSF-PB asphalt mastics. With the temperature rising, all curves increase to the maximum index and then decrease to the value that is higher than the initial flow behavior index. Secondly, with the augment of LF-SMB volume ratio, the flow behavior index at each temperature presents a decreasing trend gradually, causing that the asphalt mastics with 0.4 and 0.6 LF-SMB volume ratio cannot reach the flow state whose flow behavior index must be greater than 0.9. These phenomena can be explained as follows:

SBS polymer is a thermoplastic elastomer which consists of styrene-butadiene-styrene monomer. Polystyrene segments (S) are at both ends of monomer and gather together to form a physical cross-linked area, which gives the material sufficient strength. Polybutadiene segment (B) is in the center and forms a soft segment, which exhibits high elasticity and gives the material superior elastic properties. When the SBS particles are blended with the pure bitumen, the specific structure of the SBS molecule determines that it must absorb the light components of pure bitumen partly, and occurs physical cross-linking between them which is also regarded as swelling and recombination. SBS particles absorb these light components which can undergo the transition of aggregation states at ambient temperature, making the modified bitumen more difficult to adjust inner components to accommodate temperature variation. So that the SBS modified bitumen has lower temperature sensibility than pure bitumen and cannot reach the same flow state as pure bitumen at the identical temperature. In the initial range of temperature rising, the modified bitumen losses a portion of elastic components and the proportion of viscous components increase gradually. So the modified bitumen can have a better flow state than the beginning and the flow behavior index increases gradually at first. However, when the temperature rises to a certain value, the contribution of SBS particles, which has a superior elastic property to a shearing action, becomes more obvious. These particles can counteract the loss of elastic components and make the bitumen more viscous, finally resulting that the flow behavior index decreases after reaching the maximum. In addition, the augment of fillers even worsens the flow state and finally makes asphalt mastics with high LF-SMB volume ratio unable to reach the state as near-Newtonian liquid. In summary, because of the existence of SBS modifiers, so the flow behavior index is inadequate to evaluate the initial self-healing temperature of asphalt mastics contained SBS modified bitumen.

**Figure 10:**
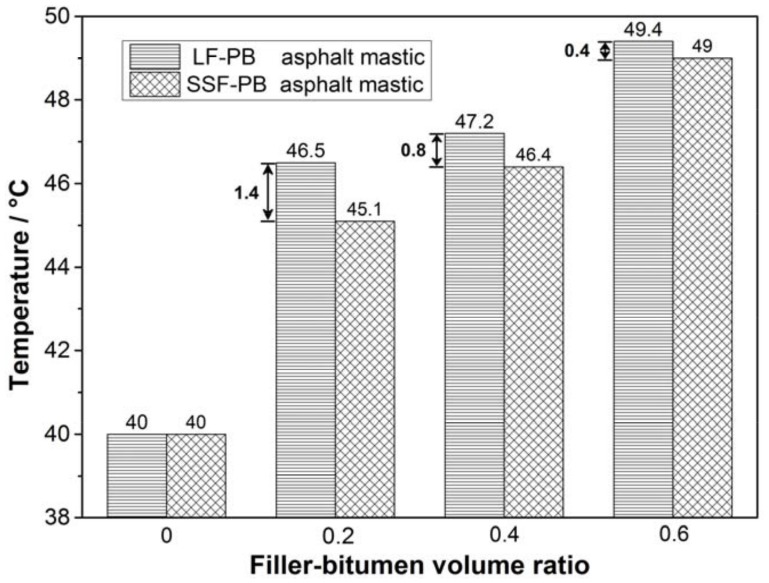
The initial self-healing temperature of different asphalt mastics.

According to Table 4 and Table 5, the initial self-healing temperatures of different asphalt mastics at which flow behavior indexes are 0.9 are further described in Figure 10. As shown in Figure 10, no matter LF-PB asphalt mastics or SSF-PB asphalt mastics, the initial self-healing temperature values present a linear growth along with the augment of filler-bitumen ratio, meaning that additions of fillers decrease the fluidity of pure bitumen and requiring a higher temperature to reach the same flow state. The initial self-healing temperature values of asphalt mastics with 0.2, 0.4 and 0.6 LF-PB volume ratio are 46.5 °C, 47.2 °C and 49.4 °C, which are 1.4 °C, 0.8 °C and 0.4 °C higher than that of asphalt mastics with 0.2, 0.4 and 0.6 SSF-PB volume ratio. The results firstly demonstrate that in the same filler-bitumen ratio, SSF-PB asphalt mastics come up to the flow characteristic more quickly than LF asphalt mastics and are prompt to heal the generated cracks easier. Secondly, although the gaps of initial self-healing temperatures between LF-PB and SSF-PB asphalt mastics exist in every same filler-bitumen ratio, the gaps show a dropping tendency that ranging from 1.4 °C to 0.4 °C. This phenomenon can be explained that in lower filler-bitumen ratio, the bitumen occupies mostly in the asphalt mastic and the better interaction between steel slag filler and bitumen can be clearly revealed in the initial self-healing temperature. However, in higher filler-bitumen ratio, the filler occupies mostly and the flow behavior of asphalt mastic is much worse, so the effect of interaction cannot be as distinct as the state of lower filler-bitumen ratio, resulting in the decrease of initial self-healing temperature gaps. Analyzed results give an overview about initial self-healing temperature of different asphalt mastic with 0–0.6 filler-bitumen ratio, in real maintenance of bituminous layer field, maintenance work can select suitable temperature based on the type of filler, bitumen and filler-bitumen ration, so that contributing to save energy and reduce energy consumption. In addition, selecting suitable temperature can delay the aging and prolong the service life of bituminous materials.

## 4. Conclusions

This research aimed to study the viscosity and flow characteristics of different asphalt mastic to determine the initial self-healing temperature. The texture and geometry characteristics of limestone filler and steel slag filler were firstly analyzed. Then the initial self-healing temperature of nine types of asphalt mastic, pure bitumen and SBS modified bitumen were evaluated by flow behavior index. According to the analyzed results, the following conclusions can be obtained:

(1) Based on the gray-scale histogram SEM images, the average gray-scale texture values of two fillers have little difference and all ranges from 110 pixels to 150 pixels. Nevertheless, the variance value of LF is around 1500 while the value of SSF is around 2500, causing that the average standard deviation value of three evaluated areas in LF is 21.24% lower than that of SSF, indicating that SSF with irregular surface texture is coarser than LF.
(2) Steel slag filler shows better form 2D value than limestone filler. The standard deviation of limestone filler is 14.56% higher than that of steel slag filler. Limestone filler has a wider distribution of angularity after being crushed, presenting more percentages in the rounded and angular categories. In summary, the steel slag filler has a better particle distribution and geometry characteristics.
(3) SBS particles can absorb light components in bitumen which can undergo the transition of aggregation states at ambient temperature, making SBS modified bitumen has lower temperature sensibility than pure bitumen and cannot reach the same flow state as pure bitumen at the identical temperature. In addition, the flow behavior indexes all increase to the maximum initially and then decrease to the value that is higher than the initial flow behavior index with the temperature rising. Because of the existence of SBS modifiers, flow behavior index is inadequate to evaluate the initial self-healing temperature of asphalt mastics contained SBS modified bitumen.
(4) The initial self-healing temperatures of asphalt mastics with 0.2, 0.4 and 0.6 LF-PB volume ratio are 46.5 °C, 47.2 °C and 49.4 °C, which are 1.4 °C, 0.8 °C and 0.4 °C higher than that of asphalt mastics with SSF-PB. It demonstrates that in the same filler-bitumen ratio, SSF asphalt mastics come up to the flow characteristic more quickly than LF asphalt mastics and are prompt to heal the generated cracks easier. Additionally, filler-bitumen ratio can also affect the initial self-healing temperature of asphalt mastic, while higher filler-bitumen ratio needs higher initial self-healing temperature to conduct self-healing process. Results show that the initial self-healing temperature can evaluate the self-healing temperature procedure. In real maintenance of bituminous layer field, maintenance work can select suitable temperature based on the type of filler, bitumen and filler-bitumen ration, so that contributing to save energy and reduce energy consumption. In addition, selecting suitable temperature can delay the aging and prolong the service life of bituminous materials.

## Figures and Tables

**Table 1:** Basic properties of raw materials.

Properties	Pure Bitumen	SBS Modified Bitumen	Properties	Limestone Filler	Steel Slag Filler
Softening point (°C)	49.4	61.7	Hydrophilic coefficient	0.70	0.66
Ductility (15 °C, mm)	>1000	>1000	Density (g/cm^3^)	2.73	3.65
Penetration (25 °C, 0.1 mm)	68.3	61.7	Water absorption (%)	0.55	0.69
Density (15 °C, g/cm^3^)	1.025	1.039	Specific surface area (m^2^/g)	1.44	1.95

**Table 2:** The statistical analysis of gray scale histogram.

Filler Classification	Limestone Filler			Steel Slag Filler		
	a1	b1	c1	a1	b1	c1
Average	120.78	111.26	132.22	117.18	141.54	117.52
Variance	1517.42	1774.89	1383.88	2502.78	2459.11	2553.74
Standard deviation	38.95	42.13	37.20	50.03	49.59	50.53

**Table 3:** The specific distribution and statistical results of tested form 2D and angularity index.

**Form 2D**						
Sample	Average	Standard Deviation	Low (≤6.5)	Moderate (6.5–8)	High (8–10.75)	Extreme (10.75–20)
Limestone filler	8.04	2.61	30.6%	29.2%	30.6%	9.7%
Steel slag filler	8.67	2.23	19.1%	24.3%	40.1%	16.4%
**Angularity Index**						
Sample	Average	Standard Deviation	Low (≤2100)	Moderate (2100–3975)	High (3975–5400)	Extreme (5400–10,000)
Limestone filler	2508.8	1040.3	40.3%	52.1%	5.6%	2.1%
Steel slag filler	2606.2	945.3	32.9%	59.2%	7.2%	0.7%

**Table 4:** Fitting results of LF-PB asphalt mastic’s flow behavior index at different temperature.

LF-PB Volume Ratio	Temperature (°C)	Fitting Formula	*n* − 1	Flow Behavior Index
0	40	y = (6 × 10^6^)x^−0.099^	−0.099	0.901
	45	y = (3 × 10^6^)x^−0.077^	−0.077	0.923
	50	y = 10^6^x^−0.058^	−0.058	0.942
	55	y = 573982x^−0.043^	−0.043	0.957
	60	y = 283129x^−0.032^	−0.032	0.968
	65	y = 145530x^−0.021^	−0.021	0.979
	70	y = 78509x^−0.015^	−0.015	0.985
0.2	40	y = (2 × 10^7^)x^−0.137^	−0.137	0.863
	45	y = (8 × 10^6^)x^−0.108^	−0.108	0.892
	50	y = (4 × 10^6^)x^−0.082^	−0.082	0.918
	55	y = (2 × 10^6^)x^−0.059^	−0.059	0.941
	60	y = 821877x^−0.043^	−0.043	0.957
	65	y = 412182x^−0.032^	−0.032	0.968
	70	y = 215878x^−0.021^	−0.021	0.979
0.4	40	y = (4 × 10^7^)x^−0.140^	−0.140	0.860
	45	y = (2 × 10^7^)x^−0.111^	−0.111	0.889
	50	y = (7 × 10^6^)x^−0.085^	−0.085	0.915
	55	y = (3 × 10^6^)x^−0.063^	−0.063	0.937
	60	y = (2 × 10^6^)x^−0.045^	−0.045	0.955
	65	y = 853056x^−0.037^	−0.037	0.963
	70	y = 446663x^−0.032^	−0.032	0.968
0.6	40	y = (4 × 10^7^)x^−0.148^	−0.148	0.852
	45	y = (2 × 10^7^)x^−0.12^	−0.120	0.880
	50	y = (7 × 10^6^)x^−0.097^	−0.097	0.903
	55	y = (3 × 10^6^)x^−0.075^	−0.075	0.925
	60	y = (2 × 10^6^)x^−0.063^	−0.063	0.937
	65	y = 899443x^−0.062^	−0.062	0.938
	70	y = 489514x^−0.06^	−0.060	0.940

**Table 5:** Fitting results of SSF-PB asphalt mastic’s flow behavior index at different temperature.

SSF-PB Volume Ratio	Temperature (°C)	Fitting Formula	*n* − 1	Flow Behavior Index
0	40	y = (6 × 10^6^)x^−0.099^	−0.099	0.901
	45	y = (3 × 10^6^)x^−0.077^	−0.077	0.923
	50	y = 10^6^x^−0.058^	−0.058	0.942
	55	y = 573982x^−0.043^	−0.043	0.957
	60	y = 283129x^−0.032^	−0.032	0.968
	65	y = 145530x^−0.021^	−0.021	0.979
	70	y = 78509x^−0.015^	−0.015	0.985
0.2	40	y = (2 × 10^7^)x^−0.131^	−0.131	0.869
	45	y = (8 × 10^6^)x^−0.101^	−0.101	0.899
	50	y = (4 × 10^6^)x^−0.077^	−0.077	0.923
	55	y = (2 × 10^6^)x^−0.056^	−0.056	0.944
	60	y = 821877x^−0.042^	−0.042	0.958
	65	y = 412182x^−0.031^	−0.031	0.969
	70	y = 215878x^−0.022^	−0.022	0.978
0.4	40	y = (4 × 10^7^)x^−0.143^	−0.143	0.857
	45	y = (2 × 10^7^)x^−0.108^	−0.108	0.892
	50	y = (7 × 10^6^)x^−0.081^	−0.081	0.919
	55	y = (3 × 10^6^)x^−0.160^	−0.060	0.940
	60	y = (2 × 10^6^)x^−0.052^	−0.052	0.948
	65	y = 853056x^−0.049^	−0.049	0.951
	70	y = 446663x^−0.047^	−0.047	0.953
0.6	40	y = (8 × 10^7^)x^−0.172^	−0.172	0.828
	45	y = (3 × 10^7^)x^−0.125^	−0.125	0.875
	50	y = 10^7^x^−0.096^	−0.096	0.904
	55	y = (7 × 10^6^)x^−0.082^	−0.082	0.918
	60	y = (3 × 10^6^)x^−0.074^	−0.074	0.926
	65	y = (2 × 10^6^)x^−0.062^	−0.062	0.938
	70	y = 866069x^−0.055^	−0.055	0.945

**Table 6:** Fitting results of LF-SMB asphalt mastic’s flow behavior index at different temperature.

LF-SMB Volume Ratio	Temperature (°C)	Fitting Formula	*n* − 1	Flow Behavior Index
0	40	y = (4 × 10^6^)x^−0.180^	−0.180	0.820
	45	y = (2 × 10^6^)x^−0.175^	−0.175	0.825
	50	y = 888198x^−0.165^	−0.165	0.835
	55	y = 465088x^−0.154^	−0.154	0.846
	60	y = 255160x^−0.149^	−0.149	0.851
	65	y = 146345x^−0.156^	−0.146	0.854
	70	y = 87869x^−0.0165^	−0.160	0.840
0.2	40	y = (7 × 10^6^)x^−0.148^	−0.148	0.852
	45	y = (3 × 10^6^)x^−0.130^	−0.130	0.870
	50	y = (2 × 10^6^)x^−0.110^	−0.110	0.890
	55	y = 808622x^−0.093^	−0.093	0.907
	60	y = 423220x^−0.083^	−0.083	0.917
	65	y = 233814x^−0.085^	−0.085	0.915
	70	y = 137396x^−0.095^	−0.095	0.905
0.4	40	y = 10^7^x^−0.162^	−0.162	0.838
	45	y = (7 × 10^6^)x^−0.145^	−0.145	0.855
	50	y = (3 × 10^6^)x^−0.123^	−0.123	0.877
	55	y = (2 × 10^6^)x^−0.106^	−0.106	0.894
	60	y = 812469x^−0.097^	−0.097	0.903
	65	y = 445488x^−0.096^	−0.096	0.904
	70	y = 269841x^−0.114^	−0.114	0.886
0.6	40	y = (2 × 10^7^)x^−0.172^	−0.172	0.828
	45	y = 10^7^x^−0.157^	−0.157	0.843
	50	y = (5 × 10^6^)x^−0.142^	−0.142	0.858
	55	y = (3 × 10^6^)x^−0.131^	−0.131	0.869
	60	y = 10^6^x^−0.118^	−0.118	0.882
	65	y = 716159x^−0.114^	−0.114	0.886
	70	y = 420473x^−0.127^	−0.127	0.873

## References

[1] Chen Z.W., Wu S.P., Xiao Y., Zeng W.B., Yi M.W., Wan J.M. (2016). Effect of hydration and silicone resin on Basic Oxygen Furnace slag and its asphalt mixture. J. Clean. Prod..

[2] Cui P.Q., Wu S.P., Xiao Y., Wan M., Cui P.D. (2015). Inhibiting effect of layered double hydroxides on the emissions of volatile organic compounds from bituminous materials. J. Clean. Prod..

[3] Gong H.R., Dong Q., Huang B.S., Jia X.Y. (2016). Effectiveness analyses of flexible pavement preventive maintenance treatments with LTPP SPS-3 experiment data. J. Transp. Eng..

[4] Robati M., Carter A., Lommerts B.J., Cotiuga I., Perraton D. New Colored Micro-surfacing Formulation with Improved Durability and Performance. Proceedings of the International Conference on Asphalt, Pevement Engineering and Infrastructure.

[5] Barcena R., Martin A.E., Hazlett D. (2002). Performance-graded binder specification for surface treatments. Transp. Res. Rec. J. Transp. Res. Board.

[6] Garcia A., Norambuena-Contreras J., Partl M.N. (2013). Experimental evaluation of dense asphalt concrete properties for induction heating purposes. Constr. Build. Mater..

[7] Demenois J., Carriconde F., Bonaventure P., Maeght J.L., Stoke A., Rey F. (2018). Impact of plant root functional traits and associated mycorrhizas on the aggregate stability of a tropical Ferralsol. Geoderma.

[8] Garcia A. (2012). Self-healing of open cracks in asphalt mastic. Fuel.

[9] Sun Y.H., Wu S.P., Liu Q.T., Li B., Fang H., Ye Q.S. (2016). The healing properties of asphalt mixtures suffered moisture damage. Constr. Build. Mater..

[10] Qiu J., van de Ven M.F.C., Wu S., Yu J., Molenaar A.A.A. (2009). Investigating the self healing capability of bituminous binders. Road Mater. Pavement.

[11] Sun D., Zhang L., Liang G. (2011). Review on self-healing behavior of asphalt concrete(1) mechanism and characterization methods of self-healing behavior. Pet. Asph..

[12] Garcia-Morales M., Partal P., Navarro F.J., Martinez-Boza F., Gallegos C., Gonzalez N., Gonzalez O., Munoz M.E. (2004). Viscous properties and microstructure of recycled eva modified bitumen. Fuel.

[13] Sung Y.T., Kum C.K., Lee H.S., Kim J.S., Yoon H.G., Kim W.N. (2005). Effects of crystallinity and crosslinking on the thermal and rheological properties of ethylene vinyl acetate copolymer. Polymer.

[14] Carreau P.J. (1997). Rheology of Polymeric Systems: Principles and Application.

[15] Heyes D.M., Mitchell P.J., Visscher P.B. (1994). Viscoelasticity and near-newtonian behaviour of concentrated dispersions by Brownian dynamics simulations. Trends in Colloid and Interface Science VIII.

[16] Li C., Chen Z.W., Wu S.P., Li B., Xie J., Xiao Y. (2017). Effects of steel slag fillers on the rheological properties of asphalt mastic. Constr. Build. Mater..

[17] Li C., Xiao Y., Chen Z.W., Wu S.P. (2017). Crack resistance of asphalt mixture with steel slag powder. Emerg. Mater. Res..

[18] Baqersad M., Hamedi A., Mohammadafzali M., Ali H. (2017). Asphalt mmixture segregation detection: Digital image processing approach. Adv. Mater. Sci. Eng..

[19] Barrett P.J. (1980). The shape of rock particles, a critical review. Sedimentology.

[20] Masad E., Olcott D., White T., Tashman L. (2001). Correlation of fine aggregate imaging shape indices with asphalt mixture performance. Geomaterials.

[21] Masad E.A. (2005). Aggregate Imaging System (AIMS): Basics and Applications.

[22] Gudimettla J., Myers L.A. AIMS: The Future in Rapid, Automated Aggregate Shape and Texture Measurement. Proceedings of the Conference of the Canadian Technical Asphalt Association.

[23] Wang H., Al-Qadi I.L., Faheem A.F., Bahia H.U., Yang S.H., Reinke G.H. (2011). Effect of mineral filler characteristics on asphalt mastic and mixture rutting potential. Transp. Res. Rec..

[24] Chen Z.W., Xie J., Xiao Y., Chen J.Y., Wu S.P. (2014). Characteristics of bonding behavior between basic oxygen furnace slag and asphalt binder. Constr. Build. Mater..

[25] Cardone F., Frigio F., Ferrotti G., Canestrari F. (2015). Influence of mineral fillers on the rheological response of polymer-modified bitumens and mastics. J. Traffic Transp. Eng..

